# Exposure to Hydroxylated Polychlorinated Biphenyls (OH-PCBs) in the Prenatal Period and Subsequent Neurodevelopment in Eastern Slovakia

**DOI:** 10.1289/ehp.0900611

**Published:** 2009-05-20

**Authors:** Hye-Youn Park, June-Soo Park, Eva Sovcikova, Anton Kocan, Linda Linderholm, Ake Bergman, Tomas Trnovec, Irva Hertz-Picciotto

**Affiliations:** 1 Division of Epidemiology, Department of Public Health Sciences, School of Medicine, University of California–Davis, Davis, California, USA; 2 California Department of Toxic Substances Control, California Environmental Protection Agency, Berkeley, California, USA; 3 Department of Toxic Organic Pollutants, Slovak Medical University, Bratislava, Slovakia; 4 Department of Environmental Chemistry, Stockholm University, Stockholm, Sweden

**Keywords:** Bayley Scales of Infant Development, hydroxylated PCBs, Slovakia

## Abstract

**Background:**

Hydroxylated polychlorinated biphenyls (OH-PCBs), unlike PCBs, are in general readily excreted yet are still detected in humans and animals. Active transport of OH-PCBs across the placenta and hydroxylation of PCBs by the fetus suggest the potential for greater impact on the fetus compared with the parent PCB compounds, but little is known about their health effects, particularly in humans.

**Objectives:**

The objective of this study was to evaluate the associations between prenatal OH-PCB exposure and neurodevelopment in children at 16 months of age in eastern Slovakia.

**Methods:**

A birth cohort (*n* = 1,134) was enrolled during 2002–2004. We analyzed six OH-PCB metabolites (4-OH-CB-107, 3-OH-CB-153, 4-OH-CB-146, 3′-OH-CB-138, 4-OH-CB-187, and 4′-OH-CB-172) in a subset of the cohort. The Bayley Scales of Infant Development were administered to the children at the 16-month follow-up visit. We developed multiple linear regression models predicting standardized scores for the Mental Development Index (MDI) and Psychomotor Development Index (PDI) from maternal (*n* = 147) and cord (*n* = 80) serum OH-PCB concentrations, adjusting for sex of child, district, HOME (Home Observation for Measurement of the Environment) score, and maternal score on Raven’s Progressive Matrices.

**Results:**

Cord 4-OH-CB-107 was significantly associated with lower MDI (β = −2.27; *p* = 0.01) and PDI (β = −4.50; *p* = 0.004). Also, maternal 4-OH-CB-107 was significantly associated with lower MDI (β = −1.76; *p* = 0.03) but not PDI. No other OH-PCB metabolites were associated with decreased PDI or MDI.

**Conclusions:**

Our findings showed a significant association of 4-OH-CB-107 with decreased MDI, which can possibly be mediated by endocrine disruption, altered neurotransmitter functions, or reduced thyroid hormone concentrations in brain.

Adverse effects of polychlorinated biphenyls (PCBs) on neurodevelopment have been well documented by growing evidence from *in vivo* and *in vitro* studies (Bemis and Seegal 2004; Carpenter et al. 2002; Ozcan et al. 2004; Seegal et al. 2005; Tan et al. 2004) and from epidemiologic studies (Darvill et al. 2000; Gladen et al. 1988; Jacobson and Jacobson 1996; Patandin et al. 1999; Rogan and Gladen 1991; Walkowiak et al. 2001). Although the mechanisms for this effect are still not well understood, one hypothesis is disruption of thyroid hormone homeostasis (Porterfield and Hendry 1998).

Over the past decade, there has been increasing research on hydroxylated PCBs (OH-PCBs), which are formed by cytochrome P450–mediated oxidation from PCBs (Letcher et al. 2000). Unlike PCBs, which are relatively stable and highly lipophilic, OH-PCBs are readily excreted. Nevertheless, several studies have observed detectable levels of OH-PCBs in both animals and humans (Bergman et al. 1994; Hovander et al. 2002; Park et al. 2007; Soechitram et al. 2004). Soechitram et al. (2004) found that OH-PCB levels in umbilical cord plasma were approximately 50% of maternal concentrations, whereas the parent PCBs concentrations in cord plasma were around 30% of maternal levels. The authors suggested that this difference may be explained by active transport of OH-PCBs across the placenta and hydroxylation of PCBs by the fetus itself, whereas transplacental transfer is the only source of fetal PCBs. In our study population (Park et al. 2007), the median cord-to-maternal ratios were 0.75 for the sum of OH-PCBs and 0.18 for the sum of PCBs from wet-weight–based concentrations. This result supports both transport across the placenta of these metabolites and the potential for greater impact on the fetus, compared with the parent PCB compounds.

Although *in vitro* and *in vivo* studies have shown adverse effects of OH-PCBs on thyroid or sex hormone homeostasis (Meerts et al. 2002, 2004a; Vakharia and Gierthy 2000), their health effects have scarcely been studied in humans. Researchers in Japan found OH-PCBs predicted higher free thyroxine (fT_4_) in neonates (Otake et al. 2007). Meerts et al. (2002) observed reduced levels of total thyroxine (T_4_) in fetal plasma and brain samples in rats after prenatal exposure to 4-OH-CB-107, one of the predominant PCB metabolites detected in humans. Also, the same research group reported developmental neurotoxicity of 4-OH-CB-107 in exposed Wistar rats, including impaired habituation and alterations in latencies to movement onset, indicative of deficits in learning and memory (Meerts et al. 2004b). Thus, competing binding affinity of OH-PCBs versus T_4_ might be a potential pathway to transport these hazardous chemicals to the brain, which may lead to subsequent impacts on neurodevelopment.

Even though the production of PCBs was prohibited in other countries beginning in the late 1970s, PCBs were manufactured by Chemko Inc., in Michalovce, a district in eastern Slovakia, until 1985. Discharges of PCBs into the environment resulted in widespread contamination of the local river and lake, providing pathways of human exposures in these areas. We launched a birth cohort study in eastern Slovakia in 2002, and followed up the children at 16 months of age to investigate the possible adverse effects of PCBs and metabolites on neurocognitive function.

In this study we evaluated associations between prenatal OH-PCB exposure and neurodevelopment in 16-month-old children in eastern Slovakia.

## Materials and Methods

### Study population

During 2002–2004, we recruited pregnant women at the time of delivery from two areas in eastern Slovakia: Michalovce, with high levels of PCBs from a chemical manufacturing plant, and Svidnik, located around 70 km to the northwest, with substantially lower average levels of PCBs. There is only one hospital in each district, where most deliveries occur. For eligibility criteria, we excluded mothers who had more than four previous births, were < 18 years of age at the time of delivery, lived < 5 years in their district, or had a major illness during pregnancy. We also excluded infants with severe birth defects. Further details on recruitment and data collection can be found elsewhere (Hertz-Picciotto et al. 2003).

Of 1,134 eligible participants, PCB measurements in maternal serum and in cord serum were available, at the time of this data analysis, for 1,076 and 469, respectively. Because of budget constraints, we measured OH-PCBs in maternal (*n* = 202) and cord sera (*n* = 92) for subsets within the cohort; we sampled randomly within strata defined by maternal PCB levels, with intensive sampling of the highest PCB levels (> 75th percentile) to increase statistical power. The resulting samples for maternal sera consisted of 42% below the 75th percentile, 20% in the 75th–85th percentile, 24% in the 85–95th percentile, and 14% above the 95th percentile. After sampling, 55 and 12 infants, respectively, did not complete the Bayley Scales, leaving 147 from maternal serum (122 from Michalovce and 25 from Svidnik) and 80 from cord serum (63 from Michalovce and 17 from Svidnik) for analysis of neurodevelopment.

Institutional review boards of University of California–Davis and the Slovak Medical University approved this study. Informed consent to all study subjects was given before participation. All data and specimens were collected only after written consent was obtained.

### Specimen collection

A trained nurse collected maternal blood specimens at delivery (~ 20 mL) using venipuncture into vacutainer tubes (S-Monovette Serum tubes; Sarstedt, Nümbrecht, Germany). To collect cord blood, the infant was held at the level of the introitus or the mother’s abdomen to prevent a significant shift of the infant’s blood volume. As soon as possible after suctioning, the cord was clamped and cut 4–5 cm from the infant’s abdomen. After the infant was dried and stabilized and the umbilical base appeared normal, an umbilical clamp was secured to the cord 1–2 cm distal to the abdominal wall, and any excess length was cut. Cord blood collection (30–35 mL) was done by the obstetrician or assisting nurse. All tubes of maternal and cord blood specimens were refrigerated at 5–10°C immediately after collection. Samples were transported to the biochemistry department of each local hospital within 2 hr for the next procedure. Serum was isolated by centrifugation (15 min at 3,000 rpm) and stored frozen at −18°C. These were transported to the Slovak Medical University in Bratislava, in thermo boxes with cooling cartridges to prevent thawing, and stored at −18°C. Aliquots of 5 mL were shipped to the University of California–Davis and stored at −80°C until analysis of OH-PCB metabolites.

### OH-PCB measurement

We have described the analytical method in a previous report (Park et al. 2007). In summary, we adapted a series of techniques for extraction of OH-PCBs from the serum and separation of substance groups: denaturation (hydrochloric acid and 2-propanol), liquid/liquid extraction (methyl *tert*-butyl ether:hexane 1:1), potassium chloride wash, and potassium hydroxide phase separation. The OH-PCBs in the sample extracts and calibration standards were methylated by adding diazomethane that was synthesized as described elsewhere (Sandau 2000). We treated the extracts with concentrated sulfuric acid (98%) followed by further cleanup on a sulfuric acid:silica gel column (1:2; 0.5 g) using dichloromethane:hexane (1:1; 10 mL) as the mobile phase. We analyzed the final extract by using gas chromatography (Agilent 6890N; Agilent Technologies, Palo Alto, CA, USA)/mass spectrometry (Agilent 5973N; Agilent Technologies) equipped with DB-5MS capillary column (30 m × 0.25 mm inner diameter, 0.25 μm film thickness; J&W Scientific, Folsom, CA, USA). 4′-OH-CB-159 (2.00 ng) and CB-209 (3.15 ng) were spiked as a recovery internal standard and an injection standard, respectively. We corrected serum concentrations of OH-PCBs by using the recoveries of the internal standard and subtraction of the quantity measured in each reagent blank analyzed with every batch. The method was initially set up in close collaboration with scientists at the Department of Environmental Chemistry at Stockholm University who started this research field. We then carried out interlaboratory comparisons with the Centers for Disease Control and Prevention (Atlanta, GA, USA) by analyzing their quality control samples: bovine serum spiked with OH-PCB congeners.

### Neurodevelopment assessment

One local psychologist in each district at the 16-month follow-up visit administered the Bayley Scales of Infant Development II (Bayley 1993), an individual assessment for evaluating development of children at 1–42 months of age, consisting of two scales: The mental scale measures cognitive development and consists of items to evaluate the child’s memory, habituation, and problem solving, whereas the psychomotor scale evaluates control of the gross and fine motor skills. We calculated Psychomotor Development Index (PDI) and Mental Development Index (MDI) scores based on the raw scores, which we then standardized with adjustment for the child’s actual age. We used the standardized scores as the outcomes of interest. All instruments used in this study were translated into the Slovak language. We videotaped 34 children in each district, and the psychologist in the other district reviewed and scored the tapes for an evaluation of reliability.

### Data collection

The data for covariates were obtained from an interview with the mother conducted by trained staff during the 5-day hospital stay and from the newborn medical record. The interview data included information on sociodemographic characteristics, past pregnancies, medical conditions, and medication history before and during the pregnancy. After delivery, each mother was assessed on the Raven’s Progressive Matrices (Raven 2002), a test of nonverbal intelligence. The respondent is asked to identify the missing segment that completes a visual geometric pattern; this task requires abstract reasoning. To assess the quality and quantity of stimulation given to children in the home environment, each psychologist also administered the Home Observation for Measurement of the Environment (HOME) instrument (Bradley and Caldwell 1984) at the clinics during the 16-month follow-up visit. HOME provides a systematic evaluation of the nurturing environment of the child based on an interview with the parent and an observation of the home by the interviewer. Because we administered HOME at the clinics rather than at the homes, we deleted three questions from the original version regarding direct observations of play materials in the home. Based on a birth cohort study from Dusseldorf, scores from this modified version were highly correlated with scores from the full scale (Winneke G, Walkowiak J, personal communication).

### Data analysis

Maternal and cord OH-PCB concentrations, the primary predictor variables of interest, were skewed (Shapiro–Wilk test, *p* < 0.0001), and hence were natural-log transformed. They were not lipid adjusted because of high water solubility of these metabolites; values are therefore expressed as nanograms per gram of serum (i.e., on a wet-weight basis).

We fitted linear regression models with the MDI or the PDI as outcomes in four separate sets of models: two for each outcome in relation to exposures from each of the two different media, maternal and cord sera. The six most abundant OH-PCB congeners—4-OH-CB-107, 3-OH-CB-153, 4-OH-CB-146, 3′-OH-CB-138, 4-OH-CB-187, and 4′-OH-CB-172—were each analyzed in relation to Bayley scores using multiple linear regression models that included covariates from a previously developed model of the Bayley Scales in which prenatal PCBs were the exposures of interest (Park et al. 2009); in that previous data analysis, MDI or PDI scores were regressed on each potential covariate in a separate model to determine the strength of the bivariate associations. Then we fit multiple linear regression models with all covariates showing a bivariate association with the PDI or MDI where *p* < 0.3 and implemented a backward elimination approach with a criterion of a change in estimate of ≥ 10% (Mickey and Greenland 1989) for inclusion of potential confounders. Covariates thereby selected for the final regression models were sex, HOME score, mother’s Raven score, and district.

In all models, we adjusted for the sampling design of stratified sampling without replacement based on strata of maternal PCB concentrations. We performed these analyses in the SUDAAN statistical package (version 9.0.2; Research Triangle Institute, Research Triangle Park, NC, USA).

## Results

Table 1 describes characteristics of the two sample subsets used in this analysis as well as the full cohort with maternal PCB measurements (*n* = 1,076). Overall, most of the basic characteristics were similar among the two subset groups and the original cohort. However, there were slight differences in some factors compared with the original cohort: More mothers with high school education, more female children, and fewer Romani subjects (an ethnic minority in Europe) were in the subset of the cohort used for the present analysis.

The arithmetic means for the sum of OH-PCB metabolites before natural log-transformation from maternal and cord sera were 0.671 and 0.497 ng/g wet weight, respectively (Table 2). Overall, OH-PCB concentrations from the cord sera were lower than those from maternal sera. 4-OH-CB-187 and 4-OH-CB-146 were, respectively, the most and second most abundant congeners in both maternal and cord sera.

Results of models for scores on the MDI, after adjustment for residential district, HOME score, sex, and Raven’s score of mother, showed no significant associations with the sum of the OH-PCB congeners either from maternal or from cord serum (Table 3). In adjusted models predicting the MDI from individual OH-PCB congeners, we observed no associations, except with 4-OH-CB-107, which was significant for maternal and for cord serum. In both cases, higher levels of this metabolite were associated with lower scores on the MDI (cord: β = − 2.27, SE = 0.87, *p* = 0.01; maternal: β = −1.76, SE = 0.80, *p* = 0.03).

Similar to results for MDI, the adjusted models of the PDI also indicated no association with the sum of OH-PCB congeners, regardless of whether these measurements were in maternal or in cord serum. The PDI was also not associated with any of the six individual OH-PCBs in maternal serum. However, in cord serum, 4-OH-CB-107 was again significantly associated with a lower PDI score (β = −4.50, SE = 1.52, *p* = 0.004). Although not statistically significant, coefficients for all cord OH-PCBs in relation to the PDI were negative; that is, higher levels were associated with poorer scores. However, this was not observed for maternal OH-PCBs.

Table 4 shows the full multiple regression models of the Bayley Scales (MDI and PDI) as a function of 4-OH-CB-107 (natural log transformed, nanograms per gram wet weight) in maternal and cord sera. The interpretation of these models is that, adjusting for confounding, an increase from the 25th to the 75th percentile of cord 4-OH-CB-107 concentration (0.008–0.033 ng/g wet weight) predicts a 6.4-point and 3.2-point reduction in PDI and MDI, respectively. For comparison, one SD for these scores in our study is equal to 14 points for MDI and 15 points for PDI. In maternal serum, the interquartile increment (0.014–0.041 ng/g wet weight) in 4-OH-CB-107 is associated with a 1.8-point decrement in MDI. Thus, the magnitude of association was larger for the cord measurements compared with the maternal measurements.

With regard to other covariates, female children had higher MDI and PDI scores compared with male children. For example, in the model from the maternal 4-OH-CB-107, girls scored approximately 7 and 4 points higher than did boys in MDI and PDI, respectively. Overall, higher HOME and maternal Raven’s scores were associated with higher MDI and PDI. District of residence was a strong predictor of PDI: Children from Svidnik showed approximately a 9-point decrement compared with children from Michalovce.

We fitted separate regression models for MDI and PDI with maternal serum data from Michalovce only (*n* = 122), the district with the more experienced psychologist. We still found a significant association of the maternal 4-OH-CB-107 with MDI, but not with PDI (MDI: β = −1.95, SE = 1.89, *p* = 0.02; PDI: β = −0.99, SE = 0.83, *p* = 0.60).

## Discussion

To our knowledge, this is the first study to investigate neurodevelopment in humans in relation to OH-PCBs. We found no association between the sum of the measured OH-PCBs in either maternal or cord serum and neurodevelopmental scores. However, cord levels of the individual metabolite 4-OH-CB-107 were significantly associated with reduced PDI and MDI, whereas maternal concentrations of this same congener showed a significant association with decreased MDI but not PDI. Thus, deficits in MDI were consistently associated with greater prenatal exposure to the specific metabolite 4-OH-PCB-107, regardless of whether maternal or cord serum was used for measurement of this compound. This is interesting because 4-OH-PCB-107 is a metabolite of both PCB-105 and PCB-118, toxicologically acting as dioxin-like and non-dioxin-like PCB congeners, but generally categorized as dioxin-like PCBs. Also, these PCB congeners are present in the original PCB product, Delor, produced at Michalovce in rather high concentrations.

*In vivo* and *in vitro* studies on animals and their tissues as well as one human study have shown adverse effects of OH-PCBs on thyroid hormones. Otake et al. (2007) observed higher neonatal fT_4_ in association with total OH-PCBs and with 4-OH-CB-187, the most abundant hydroxylated metabolite in most human studies (Park et al 2007). Rodent studies observed reduced total T_4_ levels in fetal plasma and brain samples in rats after pre-natal exposure to 4-OH-CB-107 (Meerts et al. 2002) or the PCB mixture Aroclor 1254 (Morse et al. 1996).

These observed reductions of thyroid hormones were possibly related to the strong binding affinity of OH-PCBs to transthyretin (TTR), resulting from structural similarity (Chauhan et al. 2000) of OH-PCBs to T_4_. Considering the fact that TTR can pass the placental and blood–brain barriers (McKinnon et al. 2005; Schreiber et al. 1995), OH-PCBs bound with TTR could potentially be transported to the brain, leading to subsequent impacts on neurodevelopment. TTR is a secondary thyroid hormone transport protein after thyroxine-binding globulin (TBG) (Brouwer et al. 1998) in humans, in contrast with rodents, for which TTR is the primary carrier protein. It is unclear whether the observed negative effects by 4-OH-CB-107 in our study were related to this competitive binding affinity of OH-PCB metabolites to TTR. However, Cheek et al. (1999) found that 4-OH-CB-107 did not show a high binding affinity for other thyroid hormone transport proteins such as TBG in humans.

Alteration of steroid hormone homeostasis by PCBs has been studied, and researchers suggested that estrogen-like activity might change brain dopamine concentration (Seegal et al. 1990, 1997) or aromatase activity (Woodhouse and Cooke 2004). It is known that exposure to gonadal steroids such as estrogens and androgens during early life might alter function or morphology of some areas of the brain such as the hippocampus, which is critical for learning and memory, especially in spatial domains (Schantz and Widholm 2001). Several studies demonstrating estrogenic and antiestrogenic activities of OH-PCBs (Connor et al. 1997; Kramer and Giesy 1999; Kramer et al. 1997; Matthews and Zacharewski 2000; Vakharia and Gierthy 2000) provide evidence that exposure to OH-PCBs could affect neurodevelopment by mimicking or antagonizing these hormones.

The mechanism for neurotoxicity by 4-OH-CB-107 is not known; however, anti-estrogenicity (Moore et al. 1997) or estrogenicity (Machala et al. 2004) of 4-OH-CB-107 might have the potential to affect neurodevelopment adversely. A recent *in vitro* study showed that OH-PCBs were very potent inhibitors of the human estrogen sulfotransferase (hEST) (Kester et al. 2000), which catalyzes the sulfation and inactivation of estrogens. This inhibition of an estrogen inactivator by OH-PCBs would make estradiol more available at the target tissue, resulting in estrogenic effects. Kester et al. (2000) reported 4-OH-CB-107 to be the second strongest inhibitor of hEST [half-maximal inhibitory concentration (IC_50_), 0.15–0.25 nM] among 32 OH-PCBs, and Purinton and Wood (2000) showed that ovine fetal estrogen sulfotransferase exists in brain regions such as the hypothalamus and brainstem. Based on these findings, it seems possible that 4-OH-CB-107 affects neurodevelopment by enhancing estrogen through an indirect pathway, that is, inhibition of estrogen sulfotransferase. Although 4-OH-CB-187 and 4-OH-CB-146 were considered potent hEST inhibitors (Kester et al. 2000), we did not find similar associations with these measured major OH-PCBs. These associations might be absent because the compounds are far less potent—by more than an order of magnitude—inhibitors of hEST compared with 4-OH-CB-107 (IC_50_, 6.8–30.0 and 5.8–14.0 nM, respectively, vs. 0.15–0.25 nM for 4-OH-CB-107).

Meerts et al. (2004b) also reported developmental neurotoxicity from prenatal exposure to 4-OH-CB-107 or Aroclor 1254, which they hypothesized to have been caused by disruption of dopaminergic and noradrenergic neurotransmission (Meerts et al. 2004b). Morse et al. (1996) found 4-OH-CB-107 accumulated in fetal plasma and in forebrain of rats after maternal exposure to the PCB mixture Aroclor 1254.

4-OH-CB-107 is one of the major PCB metabolites in humans (Sandau et al. 2000; Sjödin et al. 2000), formed predominantly from PCB-105 and PCB-118 via a 1,2 shift mechanism (NIH shift) of chlorine (Letcher et al. 2000; Sandau et al. 2002). PCB-105 and PCB-118 are known to be dioxin-like PCBs in chemical structure, and it is notable that Park et al. (2009) found dioxin-like PCBs (congeners 118 and 156) to be significantly associated with decreased Bayley scores, providing plausibility for the finding reported here. The non-dioxin-like PCBs did not predict 16-month Bayley MDI scores, nor did their metabolites. Some of the non-dioxin-like PCBs (138 and 153) in cord serum were relatively significant in models of the PDI, and some of the largest coefficients for OH-PCBs, besides OH-CB-107, were the OH-metabolites of these two congeners in relation to PDI scores, even though significance was not achieved. The consistently negative and large coefficients for serum OH-PCBs in relation to PDI is also striking. A study with greater power would be more definitive. Further evidence supporting metabolites as the active toxins is the more efficient transport of the OH-PCB metabolites across the placenta compared with the parent compounds (Park et al. 2007; Soechitram et al. 2004).

As to whether other chemicals can play a role as confounders, the levels of 2,3,7,8-substituted polychlorinated dibenzo-*p*-dioxins (PCDDs) and polychlorinated dibenzofurans (PCDFs) in serum samples in Michalovce and Svidnik were comparable with those found in other European countries, in contrast to the PCB levels (Kocan A, personal communication). Thus, we cannot exclude the possibility of confounding by other persistent organic pollutants (POPs) such as PCDDs, PCDFs, and polybrominated diphenyl ethers (PBDEs), but this would require that they be causally related to deficits on the Bayley scores and also correlated with PCB metabolites. However, it seems rather unlikely that these POPs are associated with only one metabolite of PCBs, 4-OH-CB-107, and not with other metabolites in the study. For PBDEs, in general, their sources would be different, so these compounds would be unlikely to correlate well with OH-PCB metabolites.

Because we tested many OH-PCBs in relation to Bayley scores, some results could be due to chance. However, the consistency of findings with regard to the single OH-PCB and the similar specificity of findings for the parent compounds would argue that these are not sporadic findings. Exploration of multiple different congeners of OH-PCBs is justified as part of a careful evaluation of a rich data set in the context of hypothesis-driven research (Rothman 1990). Moreover, the specific findings should be interpreted based on biological mechanisms, with statistical significance playing one part in the weighing of evidence.

In this study, the means ± SD of the MDI for maternal and cord groups were 93 ± 14 and 92 ± 15, respectively. In general, the standardized scores have means of 100 in the populations for which they are standardized; for example, at 15 months of age, mean ± SD is 100 ± 15 (Bayley 1993). Different ethnic backgrounds have their own traditions that may influence learning and development, so children from other cultures may not perform appropriately on an assessment such as the Bayley Scales that have been normalized with American children. The Bayley test has not been standardized in the Slovak population; therefore, some cross-cultural or language differences might have played a role in the lower MDI scores in our study (Gagnon and Nagle 2000). After consultation with a member of our scientific advisory committee and an experienced psychologist, our team in Slovakia modified certain pictures of animals and home equipment to correspond to Slovakian culture and used them in the assessment of children. Notably, a recent Japanese study also observed lower mean MDI scores (92 ± 6) (Nakajima et al. 2006) similar to those for the Slovak population. Because our research objective was to investigate possible PCB metabolite associations with Bayley scores within this study cohort, the standardization issue is less crucial. Thus, although comparisons with other populations might not be valid, the absolute scores are not the focus of our investigation because the comparisons were internal within the Slovak population.

One psychologist in each district administered the Bayley assessment in the study. For evaluation of reliability, 34 children in each district were videotaped, and the psychologist in the other district reviewed the tapes. We observed good agreement on the MDI (Pearson correlation coefficient = 0.85) between raters. However, the reliability on PDI could not be evaluated because the children frequently moved out of range of the video camera in order to complete the PDI tasks. Thus, we cannot exclude the possibility of poor reliability on PDI among psychologists in each district, which might at least partially be a hindrance to valid estimation of the association with PDI.

In addition, the mean PDI scores in the two districts differed by a substantial margin of 9 points. It is possible that poor reliability across raters as discussed above, and/or some other unmeasured variables related to district, might have caused the substantial interdistrict difference in PDI. However, even when we fitted a model using the maternal serum data only from Michalovce, the district with the more experienced psychologist, we observed no significant association with PDI, whereas the association with MDI remained.

Because neurotoxic effects caused by hazardous chemicals depend on various factors, such as the timing, concentration, and regions in the brain of exposure, it is possible that the same chemical exposure might not cause adverse effects on all domains of development (Mendola et al. 2002). Thus, prenatal 4-OH-CB-107 exposure could specifically affect MDI but not PDI.

Park et al. (2007) compared levels of major OH-PCB metabolites in human specimens from this Slovak study population with levels from four other countries: Netherlands, Canada, Sweden, and the Faroe Islands (Fängström et al. 2002; Guvenius et al. 2003; Sandau et al. 2000; Soechitram et al. 2004). The median concentration of major OH-PCB metabolites in maternal sera in the present study was 0.47 ng/g wet weight, comparable with levels observed from Faroe Island mothers with low fish consumption and higher than Swedish and north Netherlands mothers. Diets high in fish and sea mammals in Canada and in the Faroe Islands might be the source for high OH-PCBs (Park et al. 2007). Fish consumption is not, however, a major food source in the Slovak population, implying that this population in eastern Slovakia is exposed to PCBs from other sources; analysis of dietary patterns indicates that a leading contributor to PCB concentrations is pork (Sonneborn et al. 2008).

Although the original study (Park et al. 2009) is one of a few large cohort studies in the world with high-quality measurements of low-level persistent pollutants, several limitations not previously mentioned should be noted. First, the present study includes only a subset of the original cohort, which can lead to low statistical power because of the small sample size, especially for compounds with low variability. A second limitation was the refusal rate (47%) for participation in the original cohort. Selection bias can be an issue if exposed disease groups were more or less likely to be lost to follow-up than expected. However, we did not observe differences in levels of four major PCBs (congeners 138, 153, 170, and 180) between participants and those who dropped out of the study. Although selection bias is always a possibility when response rates are low, it is difficult to envision how such bias would operate to create a strong artifactual association with a single congener and none with any of the others.

In conclusion, both maternal and cord 4-OH-CB-107 were significantly associated with reduced MDI scores (*p* < 0.05), but the data were not as consistent for PDI. The correspondence with our previous findings (Park et al. 2009) that prenatal PCB-118 and PCB-156, two dioxin-like PCBs that are also parent compounds for this metabolite, predict both lower MDI and PDI scores appears to support some specificity regarding the toxic compound. However, the explanation for the discrepancy between the parent PCB-118 and its metabolite 4-OH-CB-107 with regard to the PDI score is not clear. We did not find associations between other OH-PCB congeners and the Bayley scores. In a smaller subset, we measured methylsulfonyl metabolites, but their levels were far lower than the OH-PCBs. The significant association of 4-OH-CB-107 with decreased MDI is possibly mediated by endocrine disruption, altered neurotransmitter function, or reduced thyroid hormone in the central nervous system. Further epidemiologic studies with large sample size are necessary to confirm our findings.

## Figures and Tables

**Table 1:** Characteristics of the study groups in the cohort from two districts of eastern Slovakia, 2002–2004.

	Mother (*n* = 147)	Cord (*n* = 80)	Cohort (*n* = 1,076)
Characteristic	No. (%)^a^	No. (%)^a^	No. (%)
District			
Michalovce	122 (71.6)	63 (68.7)	757 (70.4)
Svidnik	25 (28.4)	17 (31.3)	319 (29.6)

Maternal age (years)			
18–19	8 (5.1)	4 (3.5)	93 (8.6)
20–29	103 (75.5)	61 (81.0)	760 (70.6)
≥ 30	36 (19.5)	15 (15.5)	223 (20.8)

Maternal education			
Basic schooling	28 (16.8)	16 (16.3)	229 (21.3)
High school without graduation	40 (22.5)	17 (18.2)	277 (25.7)
High school with graduation	73 (58.8)	44 (62.2)	487 (45.2)
More than college/university	6 (2.9)	3 (3.3)	78 (7.3)
Missing	0 (0)	0 (0)	5 (0.5)

Sex of child			
Male	67 (41.1)	37 (40.1)	555 (51.6)
Female	80 (58.9)	43 (59.9)	521 (48.4)

Ethnicity			
Slovakian/other	121 (84.8)	68 (86.7)	821 (76.3)
Romani	22 (14.1)	11 (12.8)	228 (21.2)
Missing	4 (1.1)	1 (0.5)	27 (2.5)

Marital status			
Married or living with partner	132 (91.6)	76 (94.9)	978 (90.9)
Never married	9 (5.6)	2 (2.7)	63 (5.9)
Divorced/widowed	4 (2.3)	1 (1.9)	31 (2.8)
Missing	2 (0.5)	1 (0.5)	4 (0.4)

Maternal smoking			
No	83 (61.3)	49 (65.9)	662 (61.5)
Yes	60 (37.6)	30 (33.6)	385 (35.8)
Missing	4 (1.1)	1 (0.5)	29 (2.7)

Maternal alcohol consumption			
No	96 (62.9)	47 (57.2)	720 (66.9)
Yes	47 (36.0)	32 (42.3)	328 (30.5)
Missing	4 (1.1)	1 (0.5)	28 (2.6)

Parity			
0	66 (47.3)	37 (47.8)	446 (41.4)
1	36 (24.8)	22 (29.0)	352 (32.7)
2	30 (17.7)	15 (15.7)	186 (17.3)
3	13 (9.5)	6 (7.5)	87 (8.1)
4	1 (0.3)	0 (0)	2 (0.2)
Missing	1 (0.3)	0 (0)	3 (0.3)

Maternal hyper- or hypothyroidism history			
No	141 (96.4)	77 (95.7)	1,016 (94.4)
Yes	2 (2.5)	2 (3.8)	33 (3.1)
Missing	4 (1.1)	1 (0.5)	27 (2.5)

Maternal diabetes history			
No	143 (98.9)	79 (99.5)	1,036 (96.3)
Yes	0 (0)	0 (0)	13 (1.2)
Missing	4 (1.1)	1 (0.5)	27 (2.5)

MDI			
Mean	93.6	92.8	
SD	13.5	13.4	

PDI			
Mean	99.8	100.3	
SD	15.6	14.9	

a Weighted percentage due to the sampling design.

**Table 2:** Distributions of OH-PCBs measured in maternal and cord sera (ng/g wet weight).

	Percentile					
Congener	5th	25th	50th	75th	95th	Arithmetic mean ± SD
Maternal sera (*n* = 147)						
4-OH-CB-107	0.007	0.014	0.023	0.041	0.126	0.037 ± 0.046
3-OH-CB-153	0.017	0.041	0.063	0.099	0.265	0.086 ± 0.083
4-OH-CB-146	0.024	0.061	0.089	0.156	0.436	0.147 ± 0.222
3′-OH-CB-138	0.014	0.031	0.056	0.086	0.177	0.074 ± 0.071
4-OH-CB-187	0.053	0.106	0.175	0.309	0.801	0.273 ± 0.364
4′-OH-CB-172	0.010	0.023	0.032	0.063	0.139	0.054 ± 0.079
Sum of OH-PCBs	0.149	0.302	0.469	0.739	1.981	0.671 ± 0.805

Cord sera (*n* = 80)						
4-OH-CB-107	0.004	0.008	0.012	0.033	0.086	0.028 ± 0.045
3-OH-CB-153	0.010	0.028	0.040	0.056	0.179	0.055 ± 0.054
4-OH-CB-146	0.024	0.040	0.073	0.109	0.380	0.114 ± 0.174
3′-OH-CB-138	0.015	0.032	0.058	0.085	0.217	0.074 ± 0.075
4-OH-CB-187	0.036	0.069	0.115	0.199	0.507	0.171 ± 0.211
4′-OH-CB-172	0.010	0.023	0.058	0.085	0.217	0.055 ± 0.056
Sum of OH-PCBs	0.112	0.220	0.340	0.539	1.495	0.497 ± 0.600

**Table 3:** Estimated coefficients [β (95% confidence intervals)] of OH-PCB concentrations in multiple linear regression models predicting Bayley Scales of Infant Development in 16-month-old children from eastern Slovakia, 2002–2004.^a^

Log-transformed exposure variables	MDI	PDI
Maternal sera (*n* = 147)		
4-OH-CB-107	−1.76 (−3.34 to −0.17)*	−1.66 (−5.40 to 2.10)
3-OH-CB-153	0.56 (−1.54 to 2.66)	1.71 (−0.89 to 4.30)
4-OH-CB-146	−0.18 (−1.77 to 1.40)	1.29 (−1.55 to 4.14)
3′-OH-CB-138	0.23 (−1.81 to 2.27)	1.73 (−0.95 to 4.41)
4-OH-CB-187	−0.11 (−2.12 to 1.89)	0.90 (−1.90 to 3.70)
4′-OH-CB-172	0.46 (−1.50 to 2.42)	0.88 (−1.55 to 3.32)
Sum of six OH-PCBs	−0.13 (−2.15 to 1.90)	0.96 (−2.00 to 3.92)

Cord sera (*n* = 80)		
4-OH-CB-107	−2.27 (−4.00 to −0.54)*	−4.50 (−7.52 to −1.48)**
3-OH-CB-153	0.31 (−2.55 to 3.17)	−2.21 (−6.34 to 1.92)
4-OH-CB-146	−0.13 (−2.83 to 2.56)	−1.52 (−5.74 to 2.69)
3′-OH-CB-138	0.20 (−2.60 to 3.00)	−1.97 (−6.09 to 2.15)
4-OH-CB-187	0.17 (−2.52 to 3.07)	−1.61 (−5.99 to 2.76)
4′-OH-CB-172	0.52 (−2.07 to 3.11)	−1.21 (−4.82 to 2.40)
Sum of six OH-PCBs	−0.11 (−2.94 to 2.71)	−2.29 (−6.75 to 2.16)

Regression parameter coefficients represent one score change in PDI and MDI for a one-unit change in log*_e_* exposure concentration (ng/g wet weight).

a After adjusting for district, HOME, sex, Raven score from multiple linear regression models.

* *p* < 0.05;

** *p* < 0.01.

**Table 4:** Multiple linear regression models for effects of 4-OH-CB-107 in maternal and cord sera on the Bayley Scales (MDI and PDI) in 16-month-old children born in eastern Slovakia, 2002–2004 [β (95% confidence intervals)].

Variable	MDI	PDI
Maternal sera (*n* = 147)		
4-OH-CB-107	−1.76 (−3.34 to −0.17)*	−1.66 (−5.40 to 2.10)
District: Michalovce versus Svidnik	1.19 (−2.78 to 5.16)	−8.87 (−14.5 to −3.26)**
HOME score	0.87 (0.56 to 1.19)**	0.52 (0.03 to 1.07)*
Raven score	0.27 (0.14 to 0.40)**	0.52 (0.32 to 0.71)**
Sex: female versus male	6.99 (3.77 to 10.2)**	4.24 (−0.41 to 8.90)

Cord sera (*n* = 80)		
4-OH-CB-107	−2.27 (−4.00 to −0.54)*	−4.50 (−7.52 to −1.48)**
District: Michalovce versus Svidnik	0.75 (−4.16 to 5.66)	−9.20 (−15.43 to −2.96)**
HOME score	0.78 (0.32 to 1.24)**	−0.03 (−0.63 to 0.57)
Raven score	0.32 (0.13 to 0.51)**	0.68 (0.41 to 0.95)**
Sex: female versus male	4.96 (0.63 to 9.29)*	0.22 (−5.33 to 5.77)

Regression parameter coefficients represent one score change in PDI and MDI for a one-unit change in log*_e_* exposure concentration (ng/g wet weight).

* *p* < 0.05;

** *p* < 0.01.
